# Severe clopidogrel‐induced DRESS with eosinophilic pneumonia associated with Epstein–Barr virus reactivation

**DOI:** 10.1002/rcr2.541

**Published:** 2020-02-20

**Authors:** Yuji Inagaki, Kazunobu Tachibana, Yasushi Inoue, Takahiko Kasai, Yoshikazu Inoue

**Affiliations:** ^1^ Department of Internal Medicine National Hospital Organization, Kinki‐Chuo Chest Medical Center Osaka Japan; ^2^ Department of Anesthesiology National Hospital Organization, Kinki‐Chuo Chest Medical Center Osaka Japan; ^3^ Department of Pathology National Hospital Organization, Kinki‐Chuo Chest Medical Center Osaka Japan; ^4^ Clinical Research Center National Hospital Organization, Kinki‐Chuo Chest Medical Center Osaka Japan

**Keywords:** Clopidogrel, DRESS, eosinophilic pneumonia, Epstein–Barr virus, ventilators

## Abstract

Drug reaction with eosinophilia and systemic symptoms (DRESS) is a type of hypersensitivity drug reaction. Here, we report the case of a 78‐year‐old man who presented with a rash, fever, dry cough, and swollen parotid glands who had been prescribed clopidogrel for one year. Computed tomography showed consolidation and interlobular septal thickening with enlarged mediastinal lymph nodes. As oxygen therapy was ineffective, the patient was intubated and bronchoalveolar lavage cellular analysis showed an increase in eosinophils. Clopidogrel was discontinued and the parotid biopsy revealed periductal lymphocytic infiltration. High doses of corticosteroids were administered, and his symptoms improved. However, his symptoms recurred when clopidogrel was restarted. Skin biopsy showed mild lymphocytic infiltration in the upper dermis with vasculitis, and Epstein–Barr virus (EBV) DNA was detected in his blood and lymph node tissue. On the basis of the pathology and disease manifestations, the patient was diagnosed with DRESS. Once clopidogrel treatment ceased, his symptoms never recurred.

## Graphical abstract

We report a case of severe clopidogrel‐induced drug reaction with eosinophilia and systemic symptom (DRESS) with concomitant eosinophilic pneumonia associated with reactivation of Epstein–Barr virus (EBV). The patient was treated with systemic corticosteroids and mechanical ventilation management. DRESS can progress to severe respiratory failure, and presence of EBV or herpes virus in the serum or resected lymph node tissue is an important finding for its diagnosis.

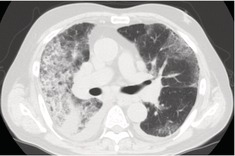

## Introduction

Drug reaction with eosinophilia and systemic symptoms (DRESS) is a potentially severe hypersensitivity drug reaction characterized by severe skin eruption, fever, lymph node enlargement, haematological abnormalities, and internal organ involvement (primarily liver disorder). This disease is associated with reactivation of the herpes virus family 1. To date, there are few reports that involve severe acute respiratory failure 2. Here, we report a case of severe clopidogrel‐induced DRESS with eosinophilic pneumonia associated with reactivation of the Epstein–Barr virus (EBV).

## Case Report

**Figure 1:**
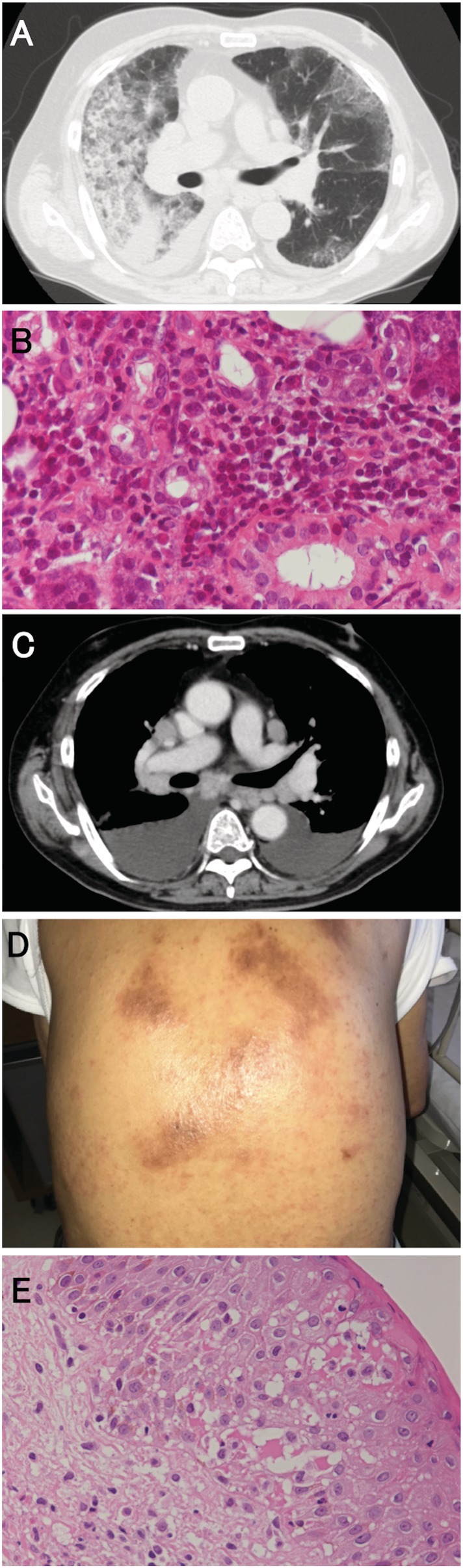
(A) Computed tomography (CT) image upon admission showing consolidation and interlobular septal thickening with enlarged mediastinal lymph nodes. (B) Histological examination of the left parotid gland revealed periductal lymphocytic infiltration haematoxylin–eosin staining 200×). (C) A CT image showing recurrent lymph node enlargement with bilateral pleural effusion. (D) The patient developed a diffuse red maculopapular rash on the trunk. (E) Histological examination of the skin showed mild lymphocytic infiltration in the upper dermis with vasculitis, associated with exocytosis of inflammatory cells in the spongiotic epidermis (haematoxylin–eosin staining 200×).

**Figure 2:**
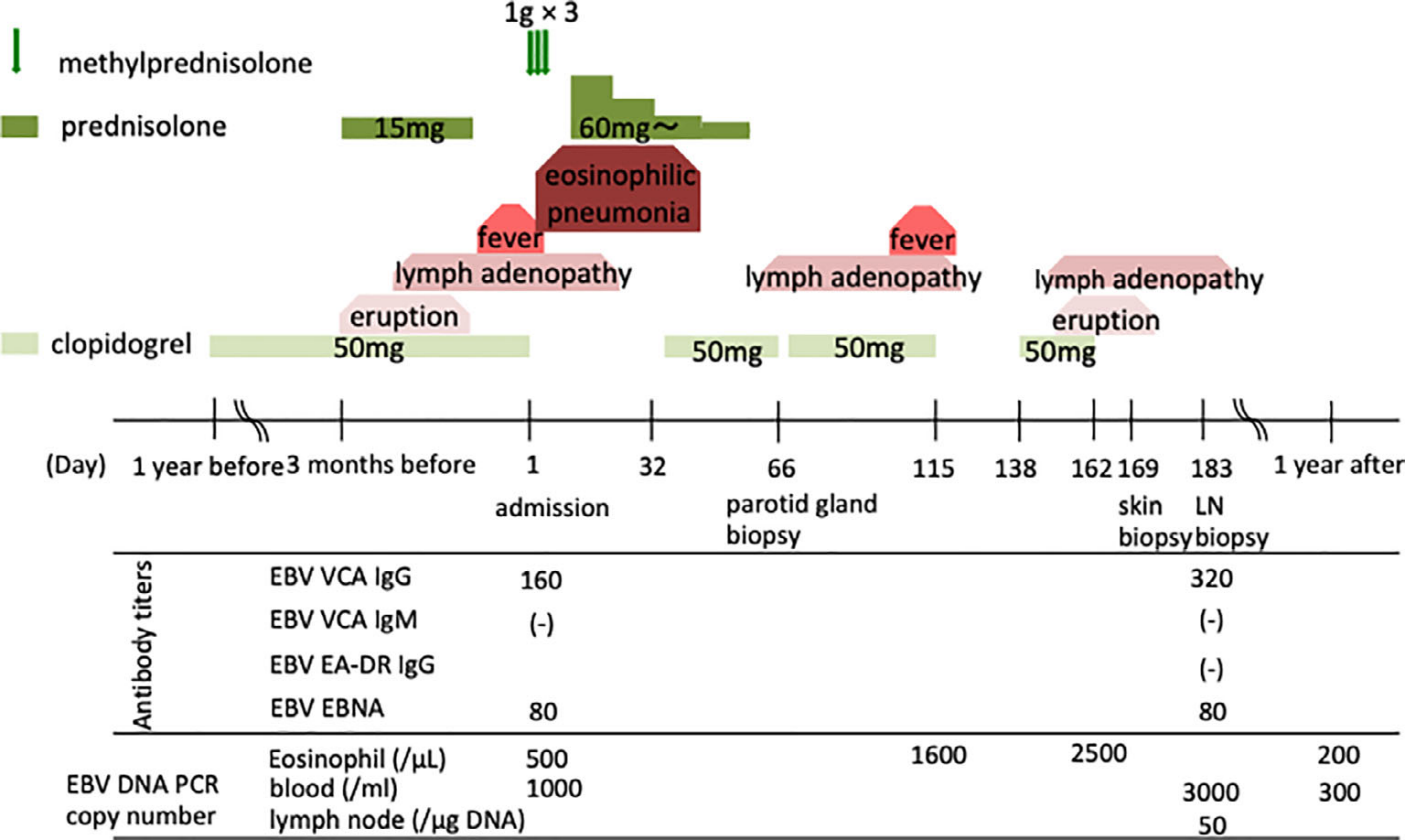
A diagram showing the clinical course. EA‐DR, early antigen‐diffuse and restricted; EBNA, Epstein–Barr virus nuclear antigen; EBV, Epstein–Barr virus; LN, lymph node; PCR, polymerase chain reaction; VCA, virus capsid antigen.

A 78‐year‐old male with a history of stroke was referred to our hospital with a fever and dry cough lasting for more than a week. He had been prescribed clopidogrel one year prior to admission. He had a rash three months before which improved with corticosteroid treatment. His body temperature was 38.6°C and his oxygen saturation of pulse oximetry (SpO_2_) was 87% on room air. A physical examination revealed fine crackles in the lower lungs and swollen parotid glands. Blood tests revealed a white blood cell count of 14,900/μL (neutrophils 75.0%, lymphocytes 10.3%, and eosinophils 11.1%). Liver function tests were normal. Computed tomography (CT) imaging showed consolidation and interlobular septal thickening with enlarged mediastinal lymph nodes and bilateral pleural effusion (Fig. 1A). As oxygen therapy was not effective, the patient was intubated and mechanical ventilation was started on the second day. Clopidogrel was discontinued considering of the possibility of a biopsy. We performed a bronchoscopy and the bronchoalveolar lavage (BAL) cellular analysis showed eosinophilia and an increase in plasma cells. A high dose (1 g) of methylprednisolone was administered for three days followed by 60 mg of methylprednisolone for the treatment of acute eosinophilic pneumonia. Oxygenation recovered and the patient was extubated on the ninth hospital day. At this time, the abnormal CT findings had improved. A parotid biopsy revealed periductal lymphocytic infiltration (Fig. 1B). His symptoms disappeared after prednisolone administration and clopidogrel was restarted on the 32nd day. After being discharged, the patient's chest symptoms recurred three months after restarting clopidogrel. Furthermore, blood tests revealed eosinophilia. Atypical lymphocytosis was found in 5% of the white blood cells. Although clopidogrel was discontinued when planning for a biopsy, his mediastinal lymph nodes got smaller without corticosteroid treatment. Clopidogrel was restarted, and three weeks later, his mediastinal lymph nodes became enlarged and bilateral pleural effusion appeared again (Fig. 1C). His rash recurred (Fig. 1D) and laboratory examination revealed elevated serum eosinophil levels of 3351/μL (34.2%). DRESS was suspected and the clopidogrel was discontinued. EBV loads were evaluated in his peripheral blood by real‐time polymerase chain reaction (PCR) and EBV viraemia was detected (EBV PCR copy number at 3000 /mL) (Fig. 2). Endobronchial ultrasound‐guided transbronchial needle aspiration (EBUS‐TBNA) of the mediastinal lymph node also detected EBV DNA (EBV PCR copy number at 50 /μg DNA) in the tissue. There was no characteristic pathological finding, such as malignancy. A skin biopsy showed mild lymphocytic infiltration in the upper dermis with vasculitis (Fig. 1E). We diagnosed him as having clopidogrel‐induced DRESS associated with reactivation of EBV. After clopidogrel treatment ceased, his symptoms did not recur.

## Discussion

DRESS symptoms usually develop within two to six weeks after drug introduction 3. DRESS diagnoses are made based on the diagnostic criteria proposed by a Japanese severe cutaneous adverse reaction group and the RegiSCAR group 1; however, clopidogrel has rarely been reported to cause this disease 4.

Although the period between taking the drug and developing the disease is longer than in previous reports, the patient met five of the former criteria (classified as an “atypical case”) and six of the latter criteria (diagnosed as DRESS). Although eosinophilic pneumonia is reported to be a complication of the disease 3, there are few reports of patients who developed severe acute respiratory failure 2. Although somewhat unclear, the incidence of lung involvement in DRESS ranges from approximately 2.6% to 5% 2. Furthermore, in a study of 15 patients with severe DRESS syndrome (mortality rate of 20%) who required treatment in an intensive care unit, 10 patients (67%) had lung complications 2. As the severity of the disease and the efficacy of systemic corticosteroids are variable and difficult to predict, a composite score for evaluating them was created 1. According to this score, this case was classified as an “8” early on, which is considered to be a “severe” case. The age of the patient and the duration of drug exposure after onset were thought to be the cause of the severity. Clinicians should carefully manage this disease as the patient's condition can worsen rapidly.

EBV or other herpes virus detection in the serum or lymph nodes is important to confirm its reactivation and diagnose DRESS. DRESS is different from other drug reactions because its pathophysiology is associated with reactivation of the herpes virus family (e.g. human herpes virus (HHV)‐6, HHV‐7, cytomegalovirus, and EBV) 1. Although reactivation is usually evaluated by the presence of bound immunoglobulin (Ig) G antibodies, real‐time PCR measurement of viral loads is considered to be another indicator 1. Detecting viral DNA using real‐time PCR in resected lymph nodes is important to assess viral reactivation in patients with DRESS 5. Thus, when DRESS is suspected, clinicians should assess reactivation with these methods.

In this report, we presented a case of severe clopidogrel‐induced DRESS with eosinophilic pneumonia associated with reactivation of the EBV. Our findings suggest that DRESS can progress to severe respiratory failure, and that EBV or other methods of herpes virus detection in the serum or resected lymph node tissue are important for its diagnosis.

### Disclosure Statement

Appropriate written informed consent was obtained for publication of this case report and accompanying images.

Y. Inagaki received personal fees from Pfizer that was unrelated to the current article. Y. Inoue reports being the advisor of and being on the committee of Boehringer Ingelheim during the time when this study was conducted, outside of the submitted work. Grants from the Japan Agency for Medical Research and Development, and the Japanese Ministry of Health, Labour, and Welfare were received outside of the submitted work.
